# Stability and Reliability of van der Waals High-κ SrTiO_3_ Field-Effect Transistors with Small Hysteresis

**DOI:** 10.1021/acsnano.5c01145

**Published:** 2025-03-19

**Authors:** Seyed Mehdi Sattari-Esfahlan, Allen Jian Yang, Rittik Ghosh, Wenwen Zheng, Gerhard Rzepa, Theresia Knobloch, Mario Lanza, Xiao Renshaw Wang, Tibor Grasser

**Affiliations:** † Institute for Microelectronics (TU Wien), Gusshausstrasse 27-29, 1040 Vienna, Austria; ‡ Division of Physics and Applied Physics, School of Physical and Mathematical Sciences, Nanyang Technological University, Singapore 637371, Singapore; § Materials Science and Engineering Program, Physical Sciences and Engineering Division, King Abdullah University of Science and Technology, Thuwal 23955-6900, Saudi Arabia; ∥ Global TCAD Solutions, 1010 Vienna, Austria; ⊥ Department of Materials Science and Engineering, National University of Singapore, Singapore 117575, Singapore; # School of Electrical and Electronic Engineering, Nanyang Technological University, Singapore 639798, Singapore

**Keywords:** single-crystal SrTiO_3_, hysteresis width, high-*k* insulators, field-effect transistors, counterclockwise hysteresis, bias temperature instability

## Abstract

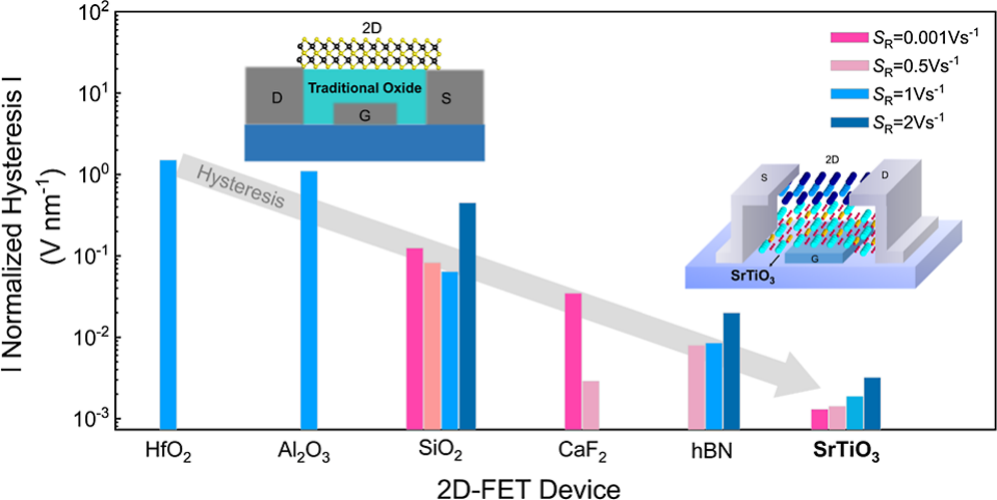

Single-crystal SrTiO_3_ (STO) is an ultrahigh-κ insulator with an expected low interface trap density that promises high breakdown strength and has great potential to boost the reliability of two-dimensional (2D) field-effect transistors (FETs). Here we provide a detailed study of the performance, stability, and reliability of MoS_2_ FETs with STO gate insulators. Most importantly, we observe a small hysteresis for electric fields up to 8 MV cm^–1^ at a sweep rate range spanning 0.01–1 V s^–1^ and sweep times of kiloseconds. Interestingly, the hysteresis is counterclockwise and bias temperature instability (BTI) is often anomalous, both likely caused by the diffusion of oxygen vacancies. We also show that the hysteresis dynamics in MoS_2_/STO FETs are reproducible over a long time, which underlines their high reliability. Our findings show that STO is a promising gate insulator that might help overcome critical obstacles to highly reliable 2D nanoelectronics.

## Introduction

Ultralow power field-effect transistors (FETs) for next-generation two-dimensional (2D) nanoelectronics demand insulators with a high dielectric constant (*k*) to minimize leakage currents and allow scaling of the physical thickness of the gate dielectric. In addition, a high-quality insulator/channel interface, a large band offset to the semiconducting channel, negligible charge trapping at the interface, and a small density of defects in the gate insulator, to enable good scalability of the equivalent oxide thickness (EOT) are desired. In this regard, various insulators including oxides,^3−5^ crystalline 2D materials,^6,7^ and amorphous insulators,^8,9^ along with oxidized 2D materials^10−12^ have been studied for being used in 2D FETs. Contrary to traditional semiconductors like silicon, a seeding layer (an initial layer needed to grow the oxide layer) is required to grow high-k oxides on 2D semiconductors, which can damage the 2D/insulator interface.^13^ A clean van der Waals interface can be fabricated by using crystalline 2D insulators such as h-BN^1^ or ionic 3D crystals like CaF_2_.^2^ Unfortunately, crystalline 2D materials such as h-BN often have a relatively small dielectric constant which eventually results in high leakage currents when the equivalent thickness is reduced below 1 nm.^14^ Strontium titanate (SrTiO_3_), STO for short, is a perovskite material with an ultrahigh dielectric constant (∼300).^15^ STO has an atomically flat surface, allowing the fabrication of multilayer 2D van der Waals heterostructures with atomically sharp interfaces.^16^ Furthermore, Yang et al. have transferred wafer-scale STO on MoS_2_ using an efficient van der Waals integration technique.^17^ Their approach addresses the structural mismatch such as poor 2D/insulator interface quality and suggests a potential route for the integration of a broad range of oxide–2D material heterostructures for FET device applications. In addition, their report demonstrated the successful fabrication of STO FET devices with MoS_2_, focusing on describing fabrication details and basic device characteristics. However, benchmarking the potential of a gate insulator material with technological importance demands an accurate understanding of its long-term stability and reliability.

Here, we evaluate the reliability of MoS_2_/STO FETs by examining changes in the transfer characteristics, the dynamics of hysteresis, and bias temperature instability (BTI) in the temperature range 76 to 400 K. We demonstrate that the crystalline STO employed in our samples produces very low counterclockwise (CCW) hysteresis and moderate anomalous BTI at all studied temperatures. Most importantly, we observe that the hysteresis does not change over time following electrical and high-temperature stress.

## Results and Discussion

### Device Structure and Initial Electrical Characterizations

**Figure 1:**
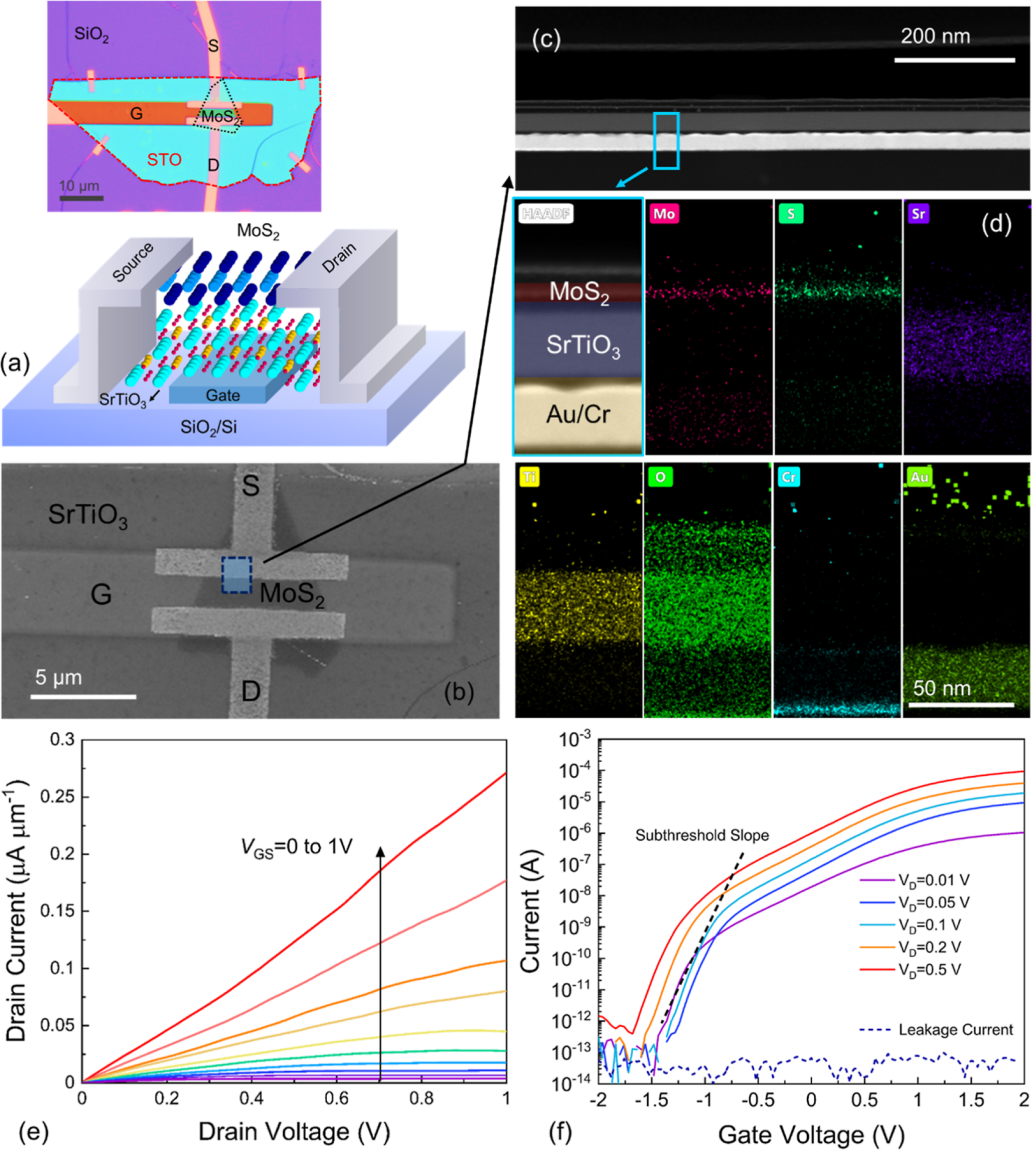
(a) Schematic of the MoS_2_/STO high-k 2D FET with bottom-gate configuration (inset: optical image). (b) A top-view image of the device taken by SEM. The cyan rectangle indicates the approximate location of the FIB cut, as shown in (c) for the HAADF-STEM, EDS, and TEM analyses. (d) The cross-sectional TEM image of the vertically stacked MoS_2_/STO/Au/Cr heterostructure on a SiO_2_/Si substrate from the area highlighted in the cyan rectangle and the corresponding EDS elemental map shows the distribution of Mo, S, Sr, Ti, O, Cr, and Au. (e) Normalized *I*_D_–*V*_D_ characteristic of the device shows saturation at lower *V*_G_. (f) Normalized *I*_D_–*V*_G_ characteristics were measured by applying different *V*_D_.

Figure 1a displays a schematic and an optical image of one of the employed bottom-gate 2D-FET with a high-k STO insulator. The device fabrication details are discussed in the Methods section and the schematics of the fabrication procedure are shown in Figure S1 (in the Supporting Information). Our MoS_2_/STO FETs have typical channel dimensions of *L* = 2.5 μm and *W* = 5 μm. However, the channel widths may vary among different devices due to the heterogeneity (diversity in size) of the exfoliated MoS_2_ flakes. The top-view layout of a device is shown in the scanning electron microscopy (SEM) image (Figure 1b). To confirm the correct fabrication of our devices, we cut a fresh device using a focused ion beam (FIB) at the location indicated with a cyan rectangle as seen in Figure 1b, and fabricated 100 nm-thick lamellae. Then, we acquired high-angle annular dark field (HAADF) cross-sectional scanning transmission electron microscope (STEM) images coupled with Energy-dispersive X-ray spectrometry (EDS) elemental maps. The images shown in Figure 1c,d confirm that the structure of the device indeed corresponds to a vertically stacked MoS_2_/STO/Au/Cr heterostructure on a SiO_2_/Si substrate. The multilayered MoS_2_ placed on the STO thin film with a physical thickness of ∼32 nm, is shown in the top-left panel of Figure 1d. The EDS maps confirm the distribution of Mo, S, Sr, Ti, O, Cr, and Au in the heterostructure. We also acquired high-resolution cross-sectional transmission electron microscopy (TEM) images of the device which confirms the correct crystalline structure of the STO film (see Figure S2).

Typical output *I*_D_–*V*_D_ characteristics measured at different *V*_G_ show promising performance with current saturation and good current control (Figure 1e). The *I*_D_–*V*_G_ characteristics of the device at different *V*_D_ are shown in Figure 1f, where *I*_D_ is normalized to the channel width (*W*). Our STO FET showed a maximum normalized current of ∼20 μA μm^–1^ (at *V*_D_ = 0.1 V), an *I*_on_/*I*_off_ ratio of 10^7,^ and a subthreshold slope (SS) value of 270 mV dec^–1^. Also, atomic-force microscope (AFM) imaging was conducted to determine the STO thickness and surface morphology. The thickness of the STO thin film was found to be ∼32 nm (see Figure S3). Also, capacitance–voltage (*C*–*V*) characteristics for metal/STO/metal (MIM) devices (Figure S4a,b) we conducted at different frequencies as shown in Figure S4c. The capacitance value was found to be constant in the frequency range of 1 kHz to 1 MHz. Based on the *C*–*V* data, the relative dielectric constants (*k*) for a few STO MIM structures were estimated to be about 26 based on a saturation capacitance of ∼0.65 pF and device area of ∼128 μm^2^. Since a low leakage current is critical for reliable FET operation, we determined the leakage current density-electric field characteristics of our MoS_2_/STO FET, see Figure S5. The device exhibited a leakage current density of ∼10^–4^ A cm^–2^ at 7.8 MV/cm at an equivalent oxide thickness of ∼4.8 nm, which is small compared to values measured on many other common gate insulators.^14,18−20^ The small leakage current of our STO is likely due to the crystalline structure which minimizes trap-assisted tunneling. However, further improvement in the processing steps, including the 2D transfer, sample cleaning procedure, and growth quality of the insulator will likely further reduce the leakage current and the observed SS of the devices.

### Transfer Characteristics and Temperature-Dependent Leakage Current

**Figure 2:**
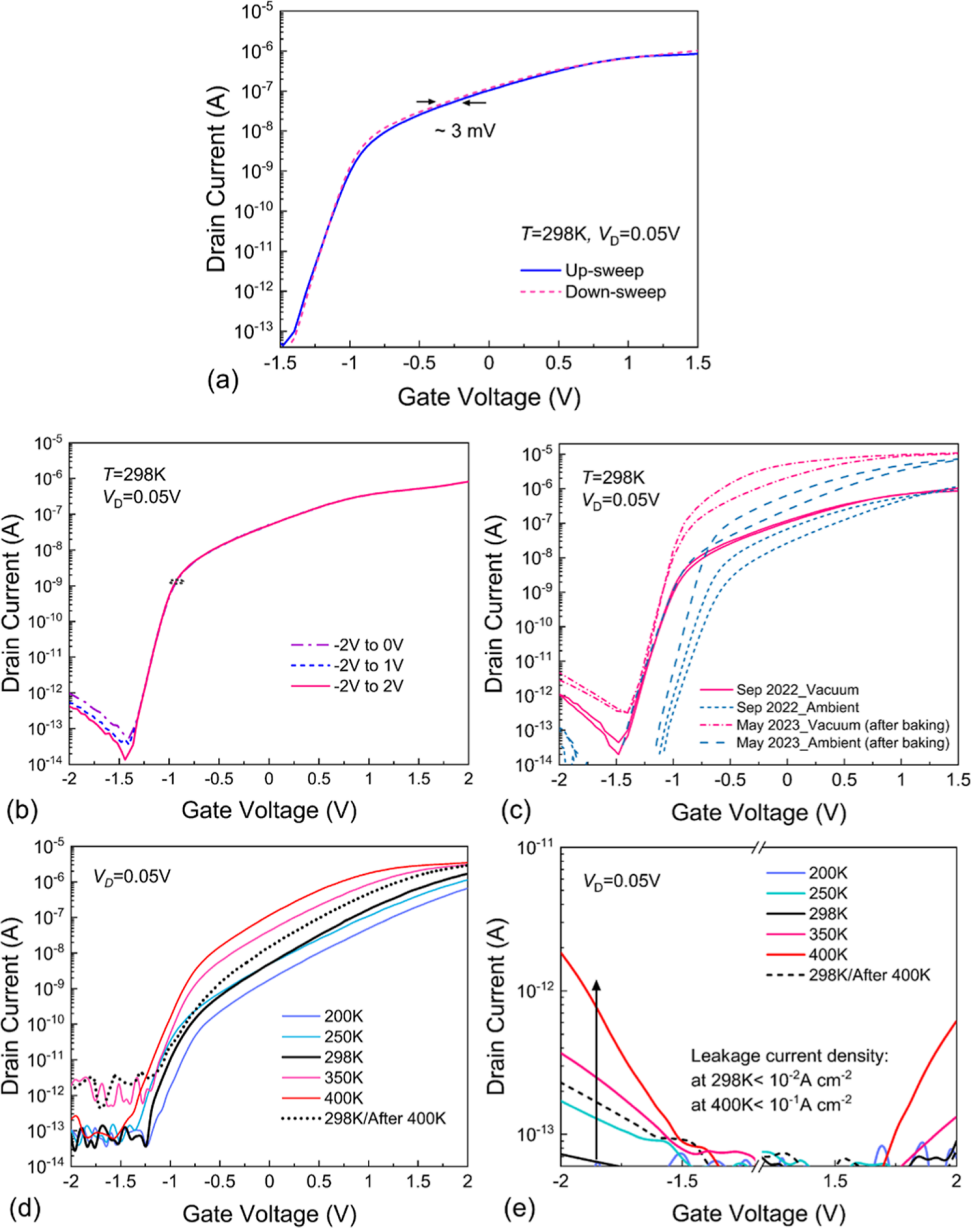
(a) Typical *I*_D_–*V*_G_ characteristics of a MoS_2_/STO FET measured in vacuum showing a small hysteresis. The *I*_D_–*V*_G_ characteristics were measured (b) using different gate voltage windows, and (c) during 8 months at vacuum and ambient conditions. (d) Temperature dependence of the *I*_D_–*V*_G_ characteristics and (e) corresponding leakage current at different *T*.

*I*_D_–*V*_G_ characteristics were measured at *T* = 298 K using both forward and backward sweeps, see Figure 2a. For a sweep rate of *S*_R_ = 0.1 V s^–1^, we observe no significant difference in *I*_D_ during up- and down-sweeps of the gate voltage, with a small hysteresis width (Δ*V*_H_) less than 3 mV. Also, we investigate the dependence of the *I*_D_–*V*_G_ characteristics measured at *T* = 298 K for different gate voltage sweep ranges at *S*_R_ = 1 V s^–1^. As shown in Figure 2b, the main device parameters such as threshold voltage (*V*_TH_) and SS are independent of the gate bias window width. Moreover, to check whether our devices age over a long time period with repetitive measurement processes both at room temperature and at higher temperatures for baking, we tracked *I*_D_–*V*_G_ characteristics during eight months of measurements followed by storage of the devices at vacuum and ambient conditions. As shown in Figure 2c, after the storage of the FETs under ambient conditions for 1 day, the threshold voltage is shifted to more positive values. Also, the hysteresis width was increased compared to the vacuum measurement, which is seemingly due to adsorbents which cause a 2× increase in hysteresis almost over the whole range of *V*_G_. However, we did not observe any significant effect on the SS and saturation current caused by ambient exposure. In addition, we measured device characteristics eight months after a high-temperature measurement at 350 and 400 K. An increase in the saturation currents can be seen for the curves repeated after eight months which may originate from the reduction of the contact resistances through the backing process in different stages of the experiments. Moreover, we did not observe a considerable threshold voltage shift of about 190 mV for our device over this eight-month storage time. Thus, a comparison of the *I*_D_–*V*_G_ curves measured over eight months proves that the device maintained its functionality over time.

The thermal activation of charge trapping, as well as impurity, and vacancy diffusion, is crucial for 2D-FET performance. Thus, understanding the impact of temperature is necessary for evaluating device behavior. We investigated the transfer curve of the device in the temperature range of 76 K to 400 K and we show some representative *I*_D_–*V*_G_ curves in Figure 2d. The critical device parameters were extracted to understand the role of temperature on the transfer characteristics. Most importantly, the drain current decreases with decreasing temperature where the saturation current reduces by almost 2 orders of magnitude when going from 400 K to 76 K. Also, the evolution of the On and Off currents as well as the threshold voltages throughout the temperature range are demonstrated in Figure S6. The On/Off current ratio was degraded at elevated temperatures and *V*_TH_ gradually shifted toward more negative values at high temperatures (see Figure S6a,b). The *V*_TH_ at room temperature is ∼0.7 V and shifts to 1.6 V at 76 K. Despite the positive shift of *V*_TH_ at low temperatures, it shifted to a negative voltage of ∼−0.25 V at 400 K. In Figure 2e we show the gate leakage current (*I*_G_) over temperature. We obtained a small leakage current *I*_G_ at *T* = 298 K which increased to ∼10^–1^ A cm^–2^ at 400 K which is still below 1 A cm^–1^ at *V*_G_ = 2 V. However, *I*_G_ is increased by about 2 orders of magnitude at 400 K, likely due to an increased thermionic emission over the relatively small barrier.

### Analysis of the Hysteresis

**Figure 3:**
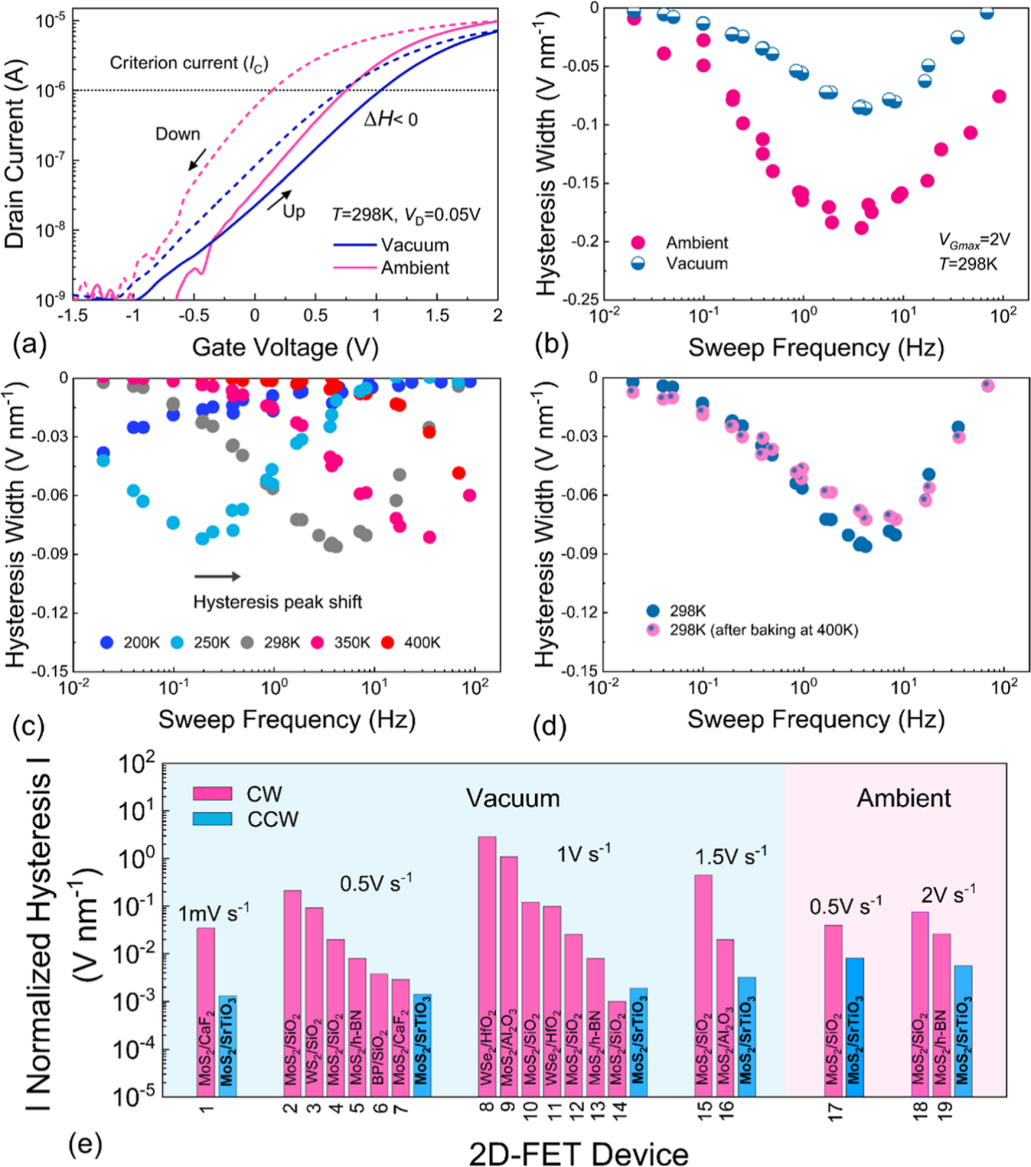
(a) The *I*_D_–*V*_G_ characteristics at vacuum and ambient conditions at *T* = 298 K. Normalized hysteresis widths Δ*V*_H_ as a function of measurement frequency extracted using a constant current criterion at (b) vacuum and ambient conditions, (c) at different temperatures measured at vacuum conditions, and (d) hysteresis width evolution after high temperature baking steps at 400 K. (e) Comparison of the absolute normalized hysteresis width measured on various 2D-FET technologies at different sweeping rates: 1, ref (2); 2, ref (25); 3, ref (29); 4, ref (30); 5, ref (31); 6, ref (32); 7, ref (2); 8, ref (33); 9, ref (34); 10, ref (35); 11, ref (33); 12, ref (7); 13, ref (7); 14, ref (7); 15, ref (36); 16, ref (36) (in vacuum); 17, ref (30); 18, ref (37); 19, ref (37) (in ambient).

To investigate the hysteresis behavior in the device, we show *I*_D_–*V*_G_ characteristics measured in a vacuum and 1 h after ambient exposure at *f*_s_ = 4 Hz as shown in Figure 3a. The sweeping frequency is *f*_s_ = 1/*t*_sw_ where *t*_sw_ is the time for one entire sweep in Figure 3b. The hysteresis width is calculated as *V*_H_ = *V*_TH,down_ – *V*_TH,up_ using a constant current criterion (*I*_C_) for the determination of the threshold voltage. Our device exhibits a small counterclockwise hysteresis width in vacuum which increases by a factor of 2 following ambient exposure. The measured hysteresis widths are normalized by the equivalent oxide thickness^21^ (EOT = 4.8 nm). Interestingly, while the majority of our devices show CCW hysteresis (Figure 3b), a single device showed clockwise (CW) hysteresis. The hysteresis values remain small for all *f*_s_ and with a maximum normalized value of ∼74 mV nm^–1^. The hysteresis width peak value is increased by about 2.5× to ∼190 mV nm^–1^ during exposure of the device to the ambient.

While CW hysteresis is typically due to charge trapping of the electrons from the channel in insulator defects, several other mechanisms have been proposed as contributors to counterclockwise (CCW) hysteresis (Δ*V*_H_ < 0) such as carrier tunneling through the traps in the MoS_2_ layer,^22^ thermal activation of oxide defects,^23^ and mobile ions in the insulator layer.^24,25^ We propose that the CCW hysteresis in our MoS_2_/STO FETs is caused by the diffusion of oxygen vacancies initially formed in the STO thin film during growth.^26,27^ A schematic illustration of the diffusion of mobile oxygen ions at different gate voltages is given in Figure S7. Also, we suggest that the increased CCW hysteresis in ambient conditions could be related to the adsorption/diffusion of oxygen and water molecules into the STO which modifies the concentration of available oxygen vacancies.

The normalized hysteresis width versus the sweeping frequency *f*_s_ at different temperatures is shown in Figure 3c. The hysteresis width is observed to be CCW at all temperatures while the peak of the hysteresis moves toward increasingly higher frequencies for elevated temperatures. The initial measurement was conducted at *T* = 298 K where a CCW hysteresis with a peak at ∼*f*_s_ = 0.1 Hz is observed. Then, the temperature was reduced to 250 and 200 K, respectively. Notably, the hysteresis peak was observed to be shifted to smaller *f*_s_ (faster sweeping rate) at lower temperatures (250 and 200 K) consistent with the fact that the mobility of the mobile ions decreases at lower temperatures. Then, we increased the temperature to 350 and 400 K, and the hysteresis peak shifted to faster sweeping frequencies. Also, to determine whether any electrochemical changes occurred in the device at these high temperatures, we measured the hysteresis width at 298 K after the measurement at 400 K as shown in Figure 3d. Importantly, the overall behavior of the hysteresis before/after the high-temperature step remained consistent which confirms recovery of the device. While virtually all devices showed CCW hysteresis, one of our devices showed CW hysteresis as a response to the same measurement procedure (Figure S8a), which is likely attributed to the higher trap density within the STO caused by variations in device processing for this particular sample. Similar to the hysteresis peak in Figure 3c, one can observe that the hysteresis peak shifts to higher frequencies as the time constants of the involved traps become smaller at higher temperatures. Also, at high sweep frequencies, the hysteresis width becomes slightly larger as the temperature is increased. However, only the left part of the hysteresis peak could be recorded in our measurements. A similar behavior was observed in previous works for MoS_2_ FETs^7,28^ with SiO_2_ and CaF_2_ as an insulator. The link of the CW hysteresis with charge trapping in STO close to the MoS_2_ interface is shown in Figure S8b. In general, a small hysteresis width is considered a critical factor for reliable device operation. Therefore, we compare the Δ*V*_H_ of our devices with various 2D FET technologies at different *V*_G_ sweeping rates and at vacuum/ambient conditions as shown in Figure 3e. Our MoS_2_/STO device shows promising hysteresis stability compared to other devices with ultralow hysteresis widths of around 2, and 9 mV nm^–1^ at vacuum and ambient conditions, respectively (e.g., at a sweeping rate of 0.5 V/s).

### Bias Temperature Instability (BTI)

**Figure 4:**
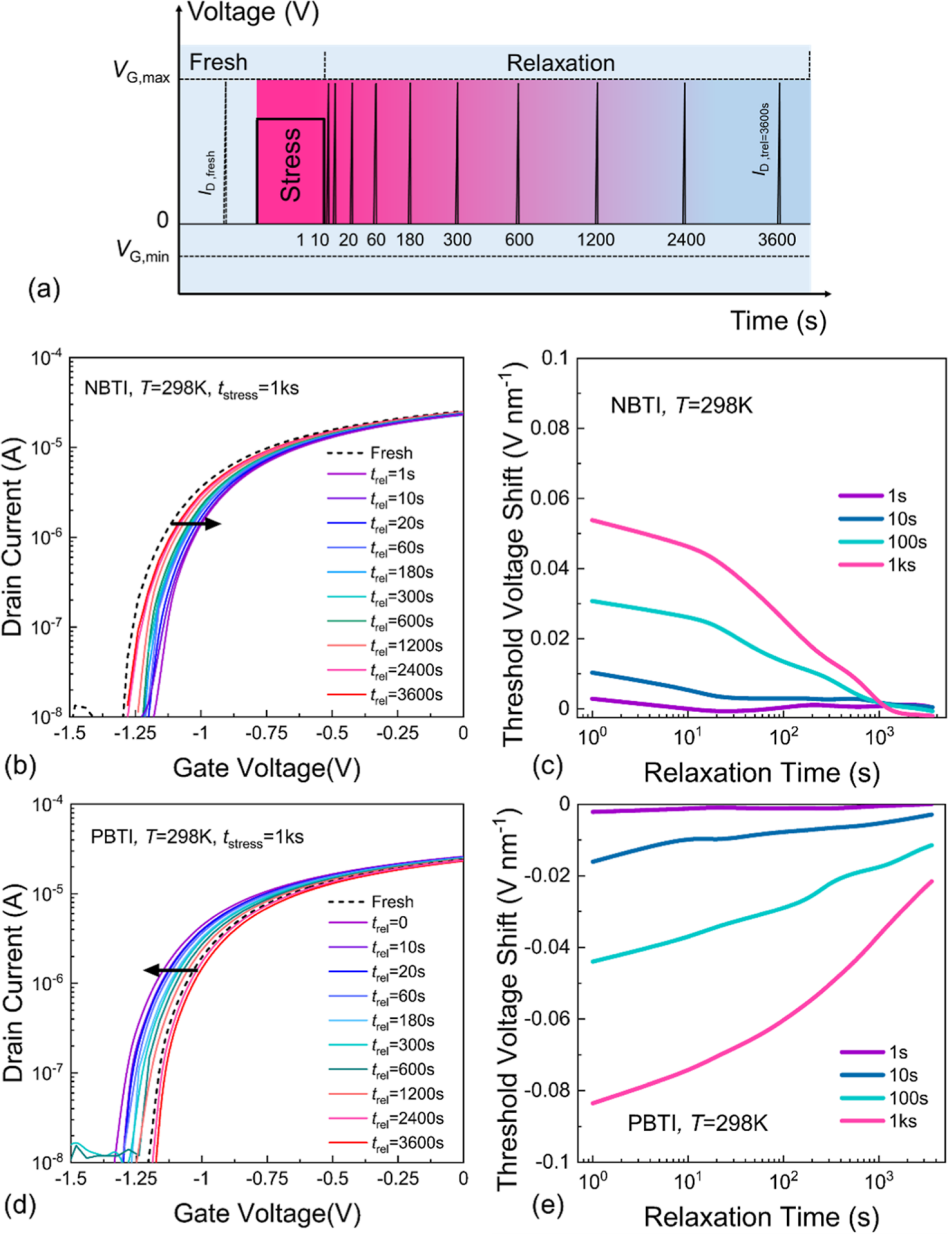
(a) A BTI measure-stress-measure procedure. Evolution of the transfer characteristics after applying subsequent BTI stresses with increasing stress times of *t*_s_ = 1 s, 10 s, 100 s, and 1 ks, under (b) negative stress bias and (c) corresponding NBTI shift, and similarly at positive stress bias (d,e) for the MoS_2_/STO FETs at *T* = 298 K.

Oxide border traps can capture and emit carriers, resulting in various instabilities.^9,34,38^ In addition to hysteresis in the transfer characteristics, the long-term drift of the threshold voltage is among the most recognized reliability challenges in 2D- and Si-FET devices, particularly due to its strong bias and temperature dependence.^19,32,39,40^ The application of gate bias stress for a certain time *t*_s_ shifts *V*_TH_ typically due to electron capture in defects moved below the Fermi level (*E*_F_). If a positive stress bias is applied, one speaks of positive BTI (PBTI) and similarly, of negative BTI (NBTI) for a negative stress bias.^41^ Note that the hysteresis width Δ*V*_H_ as discussed in the previous section is closely related to the BTI *V*_TH_ shift. Typically, the faster population of the traps contributes to the hysteresis while the slower ones to BTI.^7^ We show the employed BTI measure-stress-measure procedure in Figure 4a. First, we applied negative stress bias (*V*_G,stress_) to the device to perform NBTI measurements. We show the normalized BTI (normalized by the factor of EOT, the same as for hysteresis) results obtained by subsequent stress/relaxation cycles for different stress times (*t*_s_). Then, we measured transfer characteristics before applying any stress voltages. Next, the FET is subjected to negative gate bias stress (*V*_G,stress_) for varying stress times of *t*_s_ = 1, 10, 100, and 1 ks. After the stress period, an *I*_D_–*V*_G_ curve is measured to determine the *V*_TH_ shift using a constant criterion current. We observed a positive *V*_TH_ shift (degradation) of the *I*_D_–*V*_G_ characteristics for NBTI at higher stress times which recovers nearly completely (Figure 4b). However, the *V*_TH_ shift becomes more pronounced with increasing stress time but it is still relatively small even at a long stress time (e.g., 55 mV nm^–1^ at *t*_s_ = 1 ks and shortest recovery time *t*_rel_ = 1 s). Next, we applied a positive *V*_G,stress_ to investigate PBTI. The *I*_D_–*V*_G_ characteristics in Figure 4c show that the shift is negative for PBTI and its absolute value is larger than for NBTI. The NBTI and PBTI behavior of the device are shown in Figures 4d,e, respectively. The device shows small degradation under various PBTI stresses. Both the observed BTI degradation and the narrow hysteresis width Δ*V*_H_ at *T* = 298 K consistently indicate good device stability and reliability. Also, the recovery after NBTI stress is faster than recovery after PBTI stress. Note that the sign of the *V*_TH_ shift is different for NBTI and PBTI and that both are anomalous,^42^ that is, having a different sign of what would be expected from charge trapping. This aligns with the hysteresis measured at *T* = 298 K (Figure 3b,c) and suggests that migrating oxygen ions existing inside the STO thin film could also be responsible for this anomalous degradation. Also note that PBTI is relatively larger than NBTI at stress time *t*_s_ = 1 ks, possibly because the mobile species is in a different charge state and has a different migration barrier.

**Figure 5:**
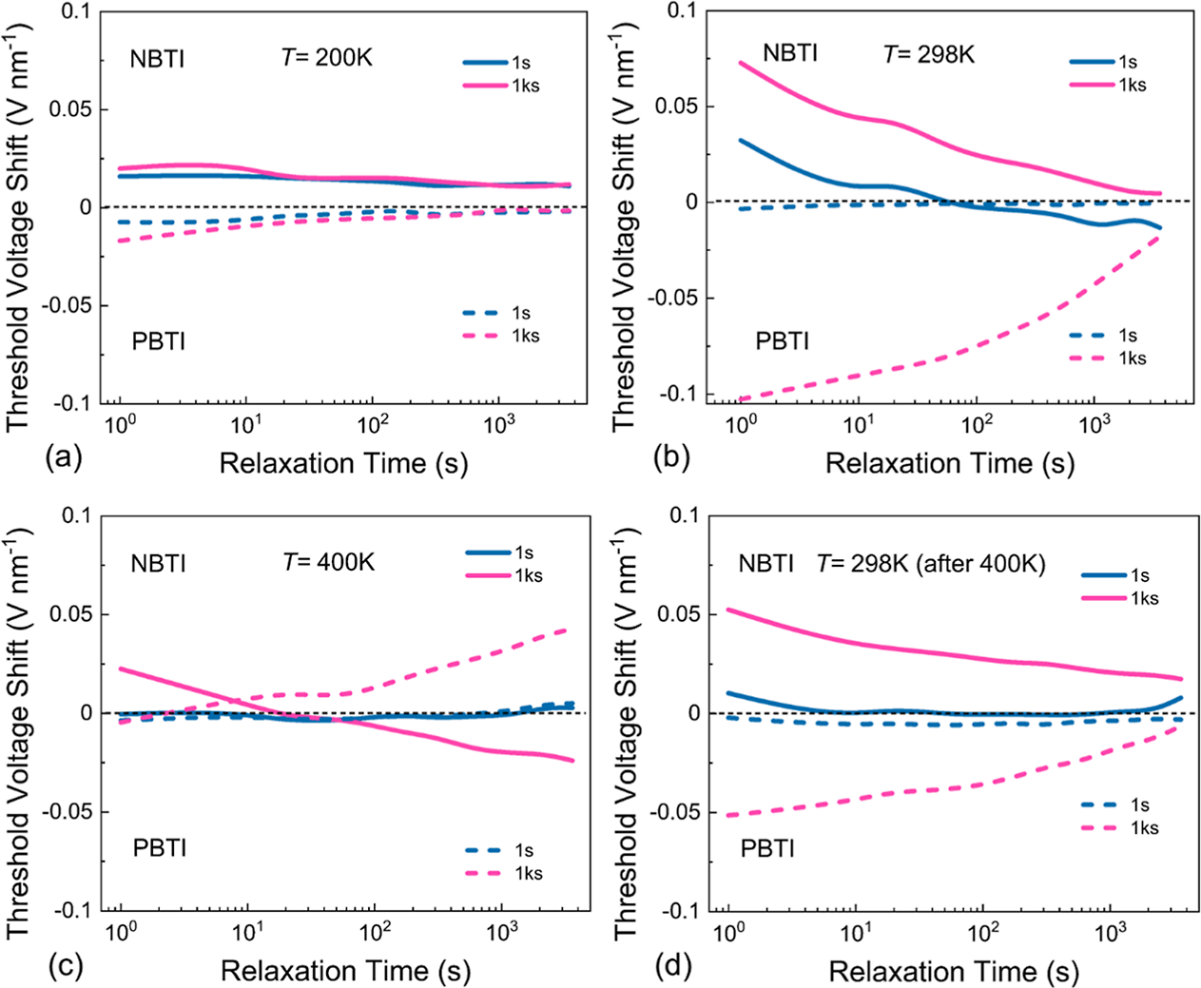
Anomalous NBTI/PBTI, at (a) *T* = 200 K, (b) *T* = 298 K, (c) *T* = 400 K, and (d) *T* = 298 K after high-temperature annealing.

Next, we analyzed the impact of temperature on the dynamics of BTI. A sequence of measurements was carried out at *T* = 200, 298, and 400 K. Then, BTI was remeasured at 298 K following the measurement at 400 K using the same measurement procedure as that of Figure 4. We show the normalized NBTI/PBTI degradation at *T* = 200 K after a stress time of *t*_s_ = 1 s and 1 ks in Figure 5a. The degradation seems to be small with an anomalous *V*_TH_ shift of <20 mV nm^–1^ for both NBTI and PBTI. This is expected because mobile ions would freeze out at low temperatures and thus not contribute to BTI. After switching back to *T* = 298 K the degradation becomes more pronounced and also again being anomalous (Figure 5b). The PBTI degradation seems to be somewhat larger than NBTI. With increasing the temperature to *T* = 400 K, BTI at short relaxation time is reduced. During recovery, both NBTI and PBTI directions change from anomalous to regular for long recovery times (Figure 5c). We argue that in addition to the mobile ions in STO, charge trapping from the MoS_2_ channel can be activated at higher temperatures. Finally, we performed a BTI test back at *T* = 298 K (10 days after performing the BTI at *T* = 400 K) and the anomalous BTI is pronounced again where there is a small difference between the *V*_TH_ shifts recorded pre- and postannealing. The device was fully recovered after NBTI degradation within about 7 h (Figure 5d). However, PBTI only partially recovers possibly due to the contribution of deeper trap levels inside the gate oxide layer or some more permeant changes in the device occurring during stress.

**Figure 6:**
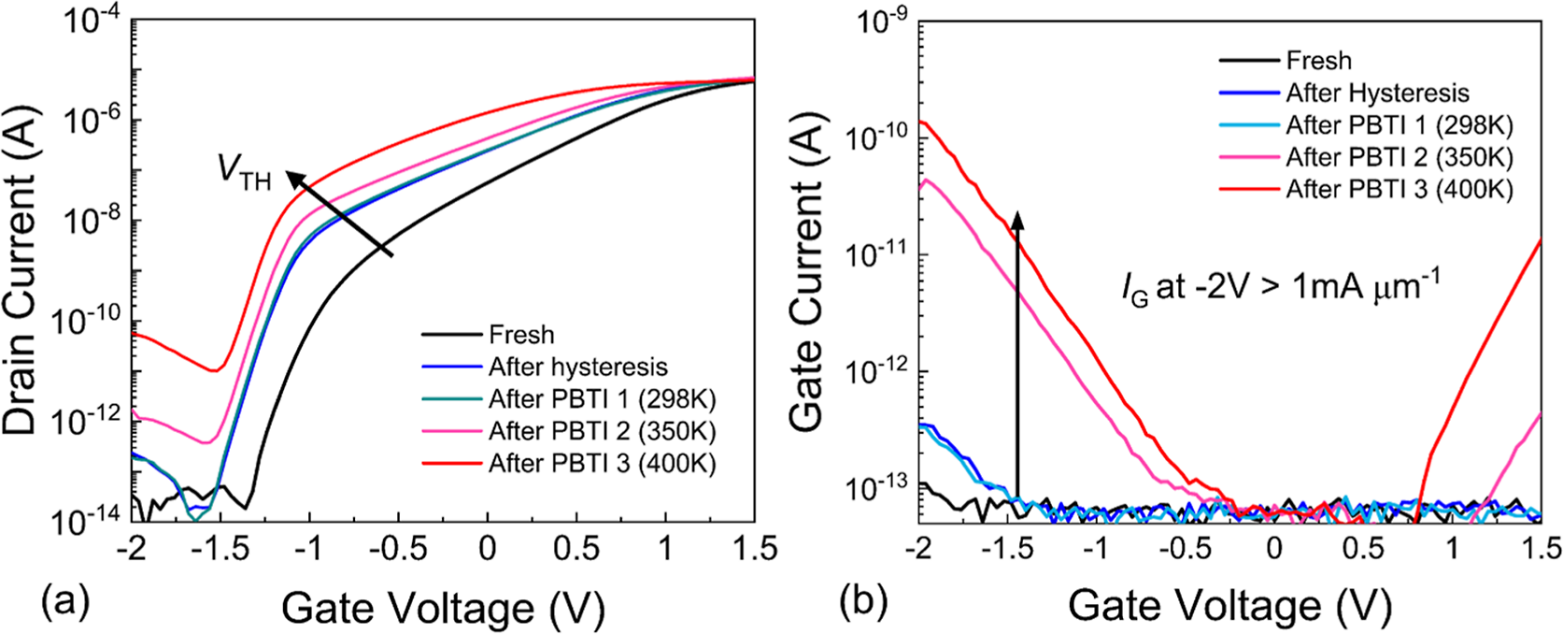
(a) Change in the *I*_D_–*V*_G_ characteristics and (b) *I*_G_–*V*_G_ characteristics after a series of hysteresis and BTI measurements.

Finally, we compare the transfer curve of our devices after each measurement step including hysteresis and frequent BTI tests as shown in Figure 6a. We observe a negative *V*_TH_ shift of the transfer curve after the sequence of hysteresis measurements compared to the fresh devices, indicating the occurrence of fixed positive charges in the STO film. While no additional changes in the *I*_D_–*V*_G_ characteristics were observed after the 298 K BTI experiment, *V*_TH_ was shifted to more negative voltages and SS degraded after PBTI tests at *T* = 350 K and *T* = 400 K. The negative shift of *V*_TH_ is related to the accumulation of positive charges in STO, possibly the creation of oxygen vacancies during the voltage stress and annealing cycles. Also, the leakage current increased by more than 2 orders of magnitude compared to the fresh device as shown in Figure 6b. However, even at high temperatures, the leakage current density remains smaller than 1 mA μm^–1^.

## Conclusions

We fabricated MoS_2_ FETs using single crystalline STO as a high-k dielectric and conducted an in-depth study on their electrical properties, hysteresis dynamics, and bias temperature instability. We observed that the MoS_2_/STO FETs had good electrical stability, most of them with a small CCW (anomalous) hysteresis. The hysteresis behavior is reproducible and remains relatively stable over 8 months. The CCW hysteresis is attributed to the diffusion of oxygen vacancies initially formed during STO growth. Also, BTI degradation is found to be anomalous which is likely also caused by mobile oxygen vacancies in the STO layer. The high long-term stability of our devices confirms that crystalline STO is a promising insulator for next-generation 2D nanoelectronics.

## Methods

### Material Preparation

The Sr_3_Al_2_O_6_ (SAO) sacrificial layer was grown by PLD on a bulk STO (001) substrate kept at 800 °C. Then, the STO film was grown at 750 °C and cooled to room temperature. The oxygen partial pressures for SAO and STO growth were 2 × 10^–5^ and 1 × 10^–1^ Torr, respectively. After growth, the STO film was lifted off by covering it with a PDMS sheet and dissolving the SAO layer in deionized water. The STO film on PDMS was aligned under an optical microscope and pressed onto the prefabricated Au gate electrodes on Si/SiO_2_ substrates. The STO film remained on the Si/SiO_2_ substrates after peeling off the PDMS sheet for the next device fabrication steps.

### Device Fabrication

The STO film was grown by the pulsed laser deposition (PLD) method on TiO_2_-terminated STO (001) substrates.^17^ The back gate electrode was fabricated on a SiO_2_/Si substrate followed by thermal deposition of 5 nm Cr and 60 nm Au. Next, the STO surface was covered by a PDMS sheet and was peeled off gently to leave the STO flake on the initially cleaned SiO_2_/Si substrate containing the fabricated back gate electrode. Then, the mechanically exfoliated MoS_2_ flake was aligned under an optical microscope and transferred to STO/SiO_2_/Si. The drain and source electrode area were determined using electron-beam lithography followed by thermal deposition of 5 nm Cr and 60 nm Au. Finally, a metal lift-off was carried out to remove extra metals and achieve drain and source electrodes.

### Material Characterization

HADDF-STEM imaging and EDX analysis were performed at 200 kV using Titan Cs probe equipment from FEI. The AFM surface profile and thickness were performed by the Reith-83 instrument.

### Electrical Measurement

Both *I*_D_–*V*_G_ and *I*_D_–*V*_D_ characteristics were performed using a Keithley 2636 parameter analyzer in the chamber of a Lakeshore vacuum probe station (∼2 × 10^–6^ Torr) in dark conditions. The hysteresis of the *I*_D_–*V*_G_ characteristics was studied by performing double sweeps at different sweep times. The BTI behavior was measured by subsequent stress/recovery tests.

## Supporting Information Available

Fabrication process, TEM images of metal/STO/metal structure, AFM, capacitance measurements, current density over electric field, and hysteresis mechanism (PDF)
