# Supplementary material for: Stability and Reliability of van der Waals High-κ SrTiO3 Field-Effect Transistors with Small Hysteresis
Source: ACS Nano. 2025 Mar 19;19(12):12288–97. doi: 10.1021/acsnano.5c01145 (PMC11966746; doi:10.1021/acsnano.5c01145)
Supplement: Supplementary file 1 — nn5c01145_si_001.pdf [file nn5c01145_si_001.pdf]

Supporting Information for:

Stability and Reliability of Van der Waals High-k $SrTiO_3$ Field-effect Transistors with Small Hysteresis

Seyed Mehdi Sattari-Esfahlan[1], Allen Jian Yang[2], Rittik Ghosh[1], Wenwen Zheng[3], Gerhard Rzepa[4], Theresia Knobloch[1], Mario Lanza[5], Xiao. Renshaw Wang[2,6], and Tibor Grasser[1*]*

[1]Institute for Microelectronics (TU Wien), Gusshausstrasse 27–29, 1040 Vienna, Austria

[2]Division of Physics and Applied Physics, School of Physical and Mathematical Sciences, Nanyang Technological University, Singapore 637371, Singapore

[3]Materials Science and Engineering Program, Physical Sciences and Engineering Division, King Abdullah University of Science and Technology (KAUST), Thuwal 23955-6900, Saudi Arabia

[4]Global TCAD Solutions, 1010 Vienna, Austria

[5]Department of Materials Science and Engineering, National University of Singapore, Singapore 117575, Singapore

[6]School of Electrical and Electronic Engineering, Nanyang Technological University, Singapore 639798, Singapore

Corresponding Authors E-mail: sattari@iue.tuwien.ac.at and grasser@iue.tuwien.ac.at

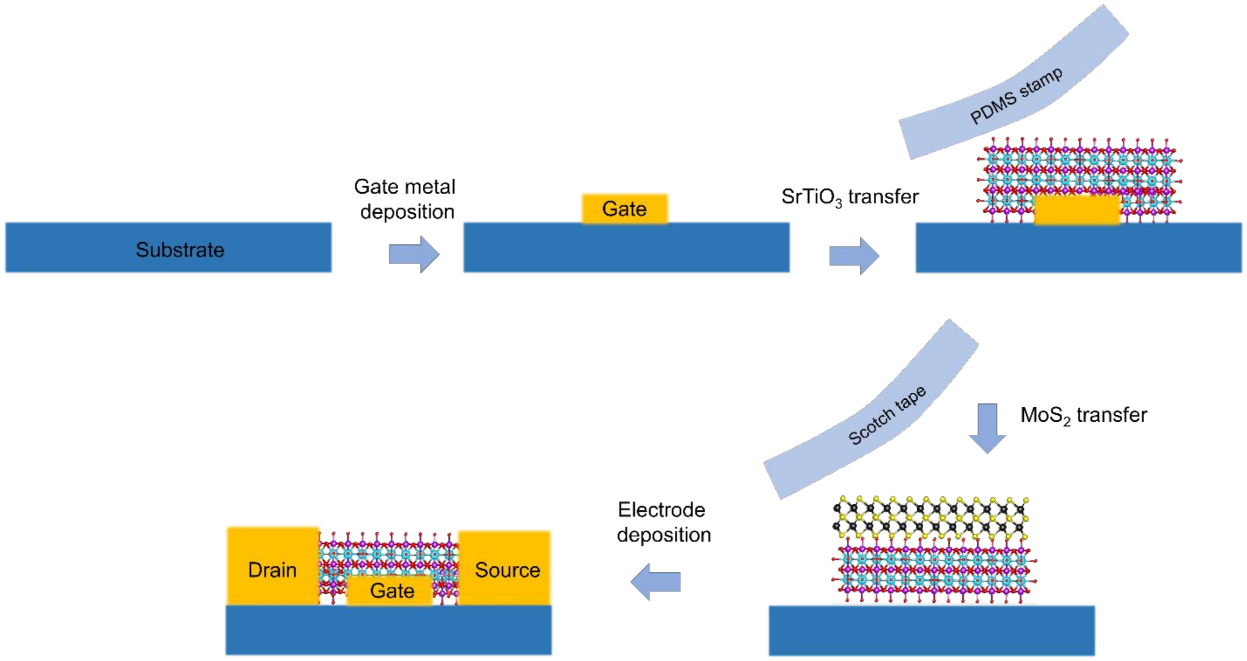

Figure S1. Schematic of the fabrication procedure of the MoS_2/STO FET devices. A PDMS sheet covers the STO, and the PDMS sheet is peeled off gently, leaving the STO layer on the initially fabricated gate electrode. Next, the drain/source electrode was fabricated, and finally, the MoS_2 layer was transferred by the scotch tape method after aligning the flake on STO.

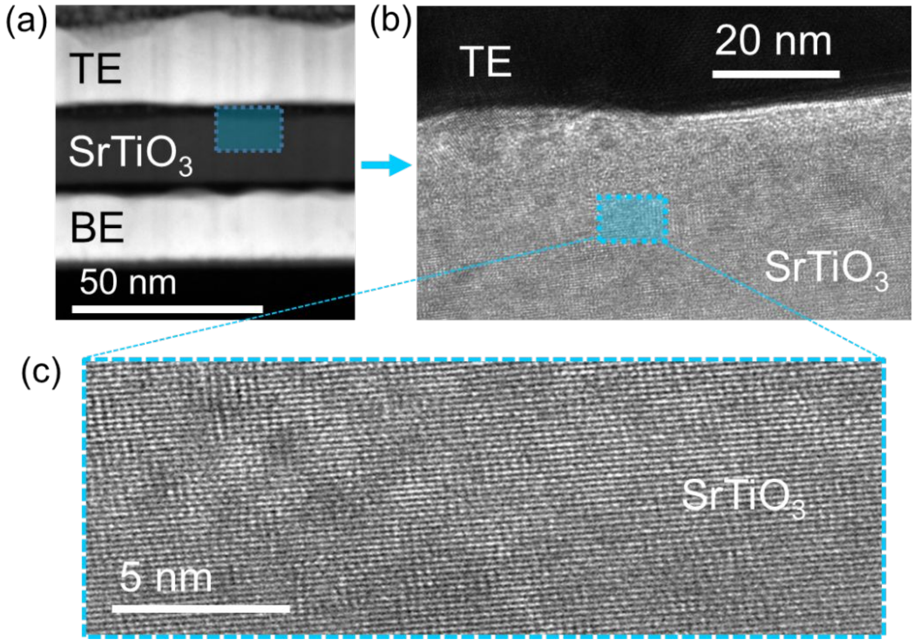

Figure S2. (a) Cross-sectional image of metal/STO /metal structure showing the STO thin film sandwiched between top and bottom metal electrodes. (b) magnified area of STO under the electrode, and (c) HR-TEM image of the STO thin film showing its crystalline structure.

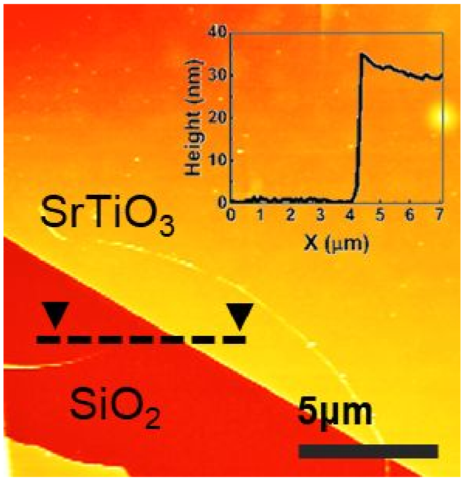

Figure S3. The surface morphology of STO on the SiO_2 substrate was recorded using AFM.

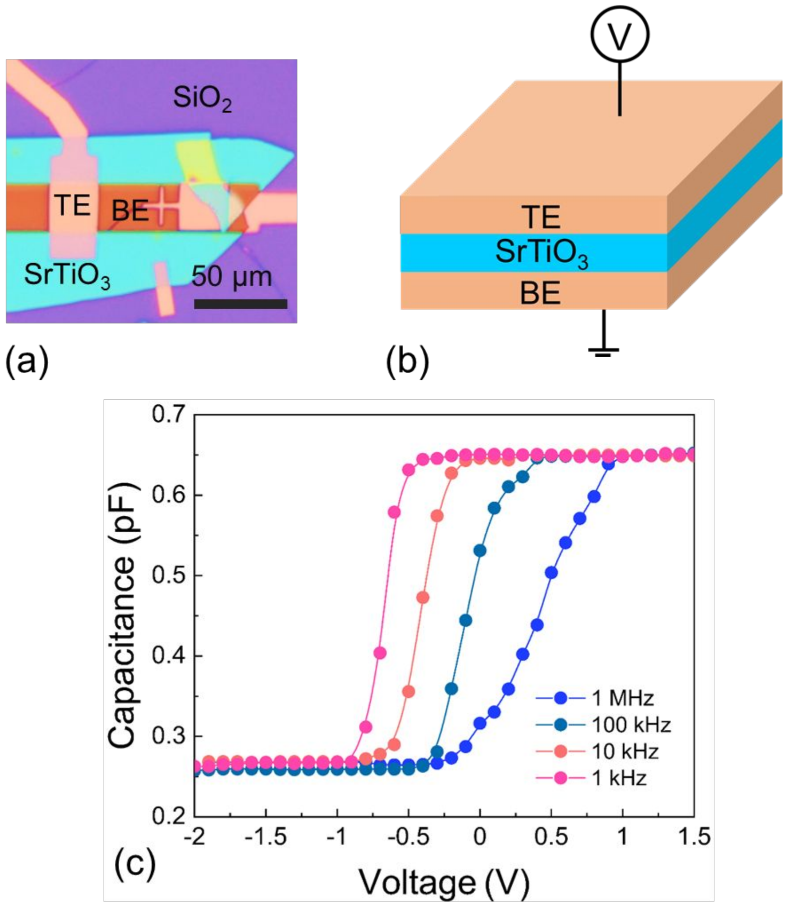

Figure S4. (a) Optical image (b) schematic of metal/STO /metal structure, and (c) capacitance-voltage characteristics

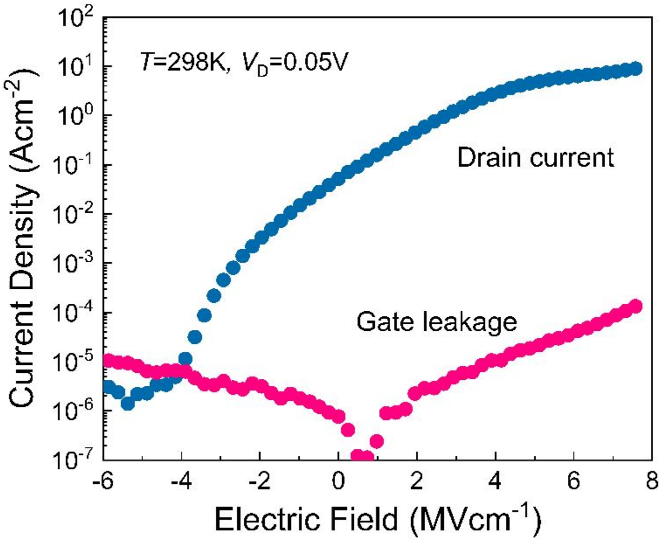

Figure S5. The drain and gate leakage current over the electric field at T=298K

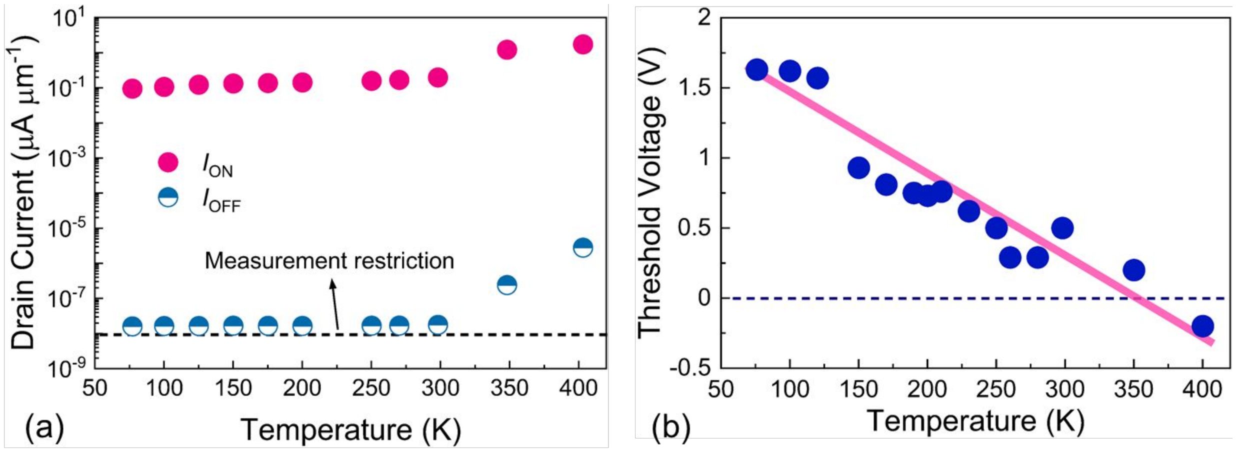

Figure S6. (a) Drain current density and (b) threshold voltage evolution over temperature for MoS_2/STO FETs

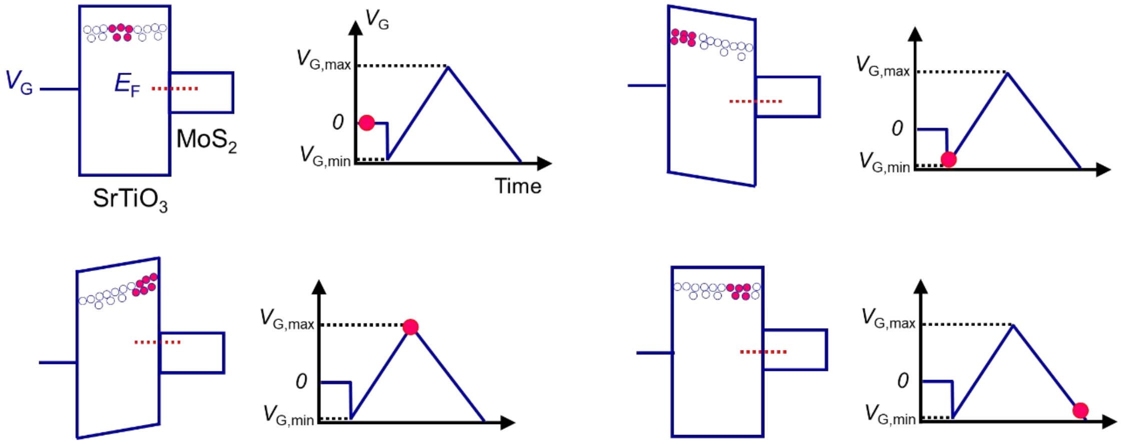

Figure S7. Mobile oxygen ions result in a negative hysteresis width (ΔV_H) peak corresponding to counter-clockwise hysteresis.

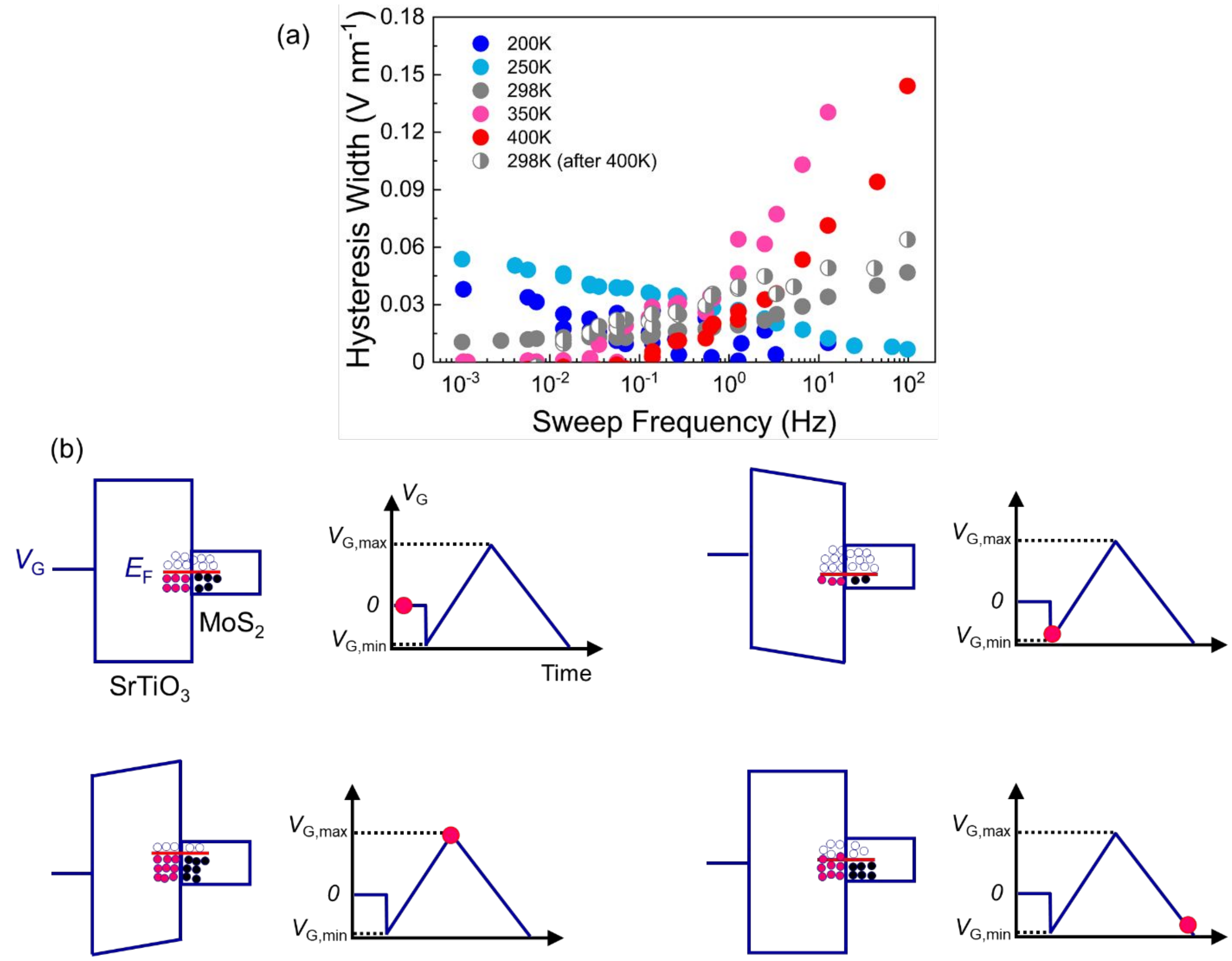

Figure S8. Normalized clockwise hysteresis widths (ΔV_H) as a function of measurement frequency at different temperatures measured at vacuum conditions. Interface traps lead to a positive ΔV_H peak, which corresponds to clockwise hysteresis.
